# Real-World Outcomes of Splenic Artery Embolization in Blunt Splenic Trauma: Insights from an Italian Multicenter Cohort

**DOI:** 10.3390/jpm15090420

**Published:** 2025-09-03

**Authors:** Fabio Corvino, Francesco Giurazza, Marcello Andrea Tipaldi, Tommaso Rossi, Francesco Daviddi, Orsola Perrone, Ilaria Ambrosini, Mauro D’addato, Ilaria Villanova, Paolo Marra, Francesco Saverio Carbone, Antonio Vizzuso, Fernando Smaldone, Anna Rita Scrofani, Roberto Iezzi, Andrea Discalzi, Marco Calandri, Marco Femia, Carlo Valenti Pittino, Ruggero Vercelli, Daniele Falsaperla, Antonello Basile, Antonio Bruno, Chiara Gasperini, Raffaella Niola

**Affiliations:** 1 Interventional Radiology Department, AORN “A. Cardarelli”, 80131 Naples, Italy; francesco.giurazza@aocardarelli.it (F.G.);; 2 Department of Surgical and Medical Sciences and Translational Medicine, Sapienza-University of Rome, 00189 Rome, Italy; 3 Department of Radiological Sciences, Oncology and Pathology, Policlinico Umberto I Hospital, Sapienza University of Rome, Viale del Policlinico 155 Sapienza, 00161 Rome, Italy; 4 UO Radiologia Interventistica, Azienda Ospedaliera Universitaria Pisa A.O.U.P., 56126 Pisa, Italy; 5 Azienda Ospedaliero-Consorziale Policlinico di Bari, P.za Giulio Cesare, 70124 Bari, Italy; 6 Department of Radiology, AAST Papa Giovanni XXIII Hospital, 24127 Bergamo, Italy; 7 School of Medicine and Surgery, University of Milano-Bicocca, 20126 Milano, Italy; 8 Radiology Unit, Morgagni-Pierantoni Hospital, AUSL Romagna, 47121 Forlì, Italy; 9 Department of Diagnostic Imaging, Oncologic Radiotherapy and Hematology, Fondazione Policlinico Universitario Agostino Gemelli IRCCS, 00168 Rome, Italy; 10 Department of Diagnostic Imaging and Interventional Radiology, City of Health and Science University Hospital, University of Turin, 10126 Turin, Italy; 11 Department of Surgical Sciences, University of Turin, 10126 Turin, Italy; 12 Interventional Radiology Unit, ASST Santi Paolo Carlo, 20122 Milan, Italy; 13 Department of Medical Surgical Sciences and Advanced Technologies “GF Ingrassia”, University Hospital Policlinico “G. Rodolico-San Marco”, 95123 Catania, Italy; 14 Diagnostic and Interventional Radiology Unit, Maggiore Hospital “C. A. Pizzardi”, 40133 Bologna, Italy

**Keywords:** splenic trauma, splenic artery embolization, non-operative management, interventional radiology, AAST grading, splenic salvage, trauma management

## Abstract

**Background:** Splenic artery embolization (SAE) has emerged as a key adjunct to non-operative management (NOM) in hemodynamically stable patients with blunt splenic trauma, yet variability in its application persists across centers. **Objectives:** The aim was to evaluate real-life clinical practices, techniques, and outcomes of SAE in blunt splenic trauma across multiple Italian trauma centers. **Materials and Methods:** This retrospective multicenter study analyzed data from 281 patients undergoing emergency SAE for blunt splenic trauma between January 2019 and December 2021. Demographics, imaging findings, embolization techniques, complications, and outcomes were collected and analyzed. Multivariate logistic regression was used to assess predictors of splenectomy. **Results:** The technical success rate was 100%, with a 9.6% rate of post-embolization splenectomy and a complication rate of 24.9% (including 5.7% splenic infarction and 3.2% rebleeding). Embolization was performed proximally (46.6%), distally (28.8%), or with a combined approach (24.6%). No significant correlation was found between embolization technique and splenectomy rate. Patients with AAST grade III injuries benefited from SAE with high technical success and low failure rates. Notably, 14.2% of patients underwent angiography despite negative CT, with a splenectomy rate of 10% in this subgroup. Multivariate analysis identified no independent predictors of splenectomy. **Conclusions:** SAE is a reliable and effective tool in the management of blunt splenic trauma, achieving high splenic salvage rates even in selected grade III injuries and CT-negative patients. In an era of precision medicine, interventional radiology should be regarded as a distinct and specific treatment modality, comparable to surgery, rather than being merely included within non-operative management (NOM).

## 1. Introduction

Splenic trauma is one of the most common intra-abdominal injuries encountered in blunt trauma cases, with the spleen being the most frequently affected solid organ. Over the past decades, the management of splenic trauma has significantly evolved, shifting from an exclusively surgical approach to a predominantly non-operative management (NOM) strategy. This transition has been driven primarily by a deeper understanding of the spleen’s immunological functions and the associated risks of splenectomy, notably overwhelming post-splenectomy infections (OPSIs). Consequently, splenic artery embolization (SAE) has become a crucial adjunctive procedure in NOM, enhancing the preservation of the spleen in hemodynamically stable patients, particularly those with high-grade injuries [1,2].

According to the latest guidelines from the World Society of Emergency Surgery (WSES), SAE is recommended in patients with AAST grade III–V splenic injuries who are hemodynamically stable or transient responders [3]. Previous studies have consistently demonstrated that SAE significantly reduces the need for surgical intervention while maintaining high rates of splenic salvage. Despite widespread adoption, considerable variability persists in clinical practice, particularly regarding embolization techniques [4,5]. Distal SAE is usually indicated for focal vascular injuries, such as pseudoaneurysms and active extravasation, whereas proximal SAE aims primarily to reduce arterial pressure and facilitate hemostasis without inducing extensive infarction, relying on collateral circulation to maintain splenic viability. Nonetheless, there remains an ongoing debate as to whether proximal embolization should be routinely performed in AAST grade IV injuries or selectively applied in grade III cases [6,7,8].

In Italy, clinical practice regarding SAE in splenic trauma appears to be heterogeneous, and assessing a realistic national splenectomy rate is currently challenging due to the lack of readily accessible and centralized data, which stems from the absence of a national trauma registry dedicated to interventional procedures. Therefore, evaluating real-life outcomes and identifying common practice patterns through multicenter Italian data is essential. This multicenter, retrospective observational study aims to provide a comprehensive analysis of SAE across several major Italian trauma centers, specifically assessing embolization techniques, clinical outcomes, and factors influencing the rate of splenectomy. By presenting real-world evidence from diverse Italian centers, this study seeks to establish a baseline understanding of current clinical practice, highlight critical trends, and contribute valuable data for future standardization efforts and optimization of SAE protocols at a national level.

## 2. Materials and Methods

### 2.1. Patient Selection and Data Collection

All patients who underwent emergency SAE following blunt abdominal trauma between January 2019 and December 2021 were included in this study. This study focused on patients who underwent SAE for blunt splenic trauma; individuals managed with primary splenectomy or other non-interventional approaches were not included, as they were outside the scope of the analysis. The cohort was composed of consecutive patients from 11 Italian trauma centers, identified in the Author Affiliations Section. Table 1 summarizes the distribution of patients and key demographic characteristics across the participating centers. Each center contributed retrospectively collected cases managed with emergency SAE between January 2019 and December 2021, using a standardized data collection protocol. The study period (January 2019–December 2021) was chosen to ensure a uniform retrospective data window across participating centers, allowing complete in-hospital outcome assessment. All submitted data were centrally curated at the coordinating center (A.O.R.N. A. Cardarelli, Naples). Manual verification was performed to identify and correct inconsistencies, missing values, or outliers through direct communication with participating centers prior to analysis. The data collection period spanned from January 2019 to December 2021. Data curation and quality control were performed between early 2022 and mid-2023, followed by statistical analysis and manuscript drafting. The final submission of the study occurred in 2025. Data collected included patient age, sex, trauma date, angiography date, and time elapsed between trauma and embolization. A standardized Excel-based data collection form was distributed to each participating center to ensure uniformity (Supplementary Table S1). Centralized curation of data was performed, including manual verification of outliers and correction of any missing or implausible values prior to analysis.

### 2.2. Radiological and Procedural Parameters

All patients underwent contrast-enhanced computed tomography (CT) prior to digital subtraction angiography (DSA). The presence of vascular injuries, such as active contrast extravasation or pseudoaneurysm, was classified as either present or absent. Splenic injury severity was assessed according to the 2018 AAST grading system. Hemoperitoneum was categorized as grade I (localized, e.g., perisplenic, perihepatic, Morrison’s pouch), grade II (extension to paracolic gutters), or grade III (pelvic involvement). SAE was classified according to the embolization site as proximal, distal, or combined. Embolic materials were documented and included pushable or detachable coils (0.035″ or 0.018″), vascular plugs, gelatin sponge (Spongostan), glue, and polyvinyl alcohol (PVA) particles. However, data concerning embolic materials were highly heterogeneous, reflecting significant variability among centers due to differing local expertise. Therefore, an accurate and detailed evaluation of embolic materials was not feasible in this analysis. Due to this heterogeneity and lack of standardized protocols, embolic materials were excluded from comparative outcome analysis.

### 2.3. Outcome Definitions

Technical success was defined as the absence of vascular lesions or active bleeding at the post-embolization angiographic control. Complications were classified according to the Cardiovascular and Interventional Radiological Society of Europe (CIRSE) guidelines and included splenic infarction, rebleeding, splenectomy, and non-target embolization [9]. Splenectomy was defined as any surgical removal of the spleen following embolization, with the time from embolization to surgery recorded in hours.

### 2.4. Statistical Analysis

Descriptive statistics, including the mean, standard deviation (SD), median, interquartile range (IQR), and proportions, were calculated for all demographic, clinical, radiological, and procedural variables. Relationships between embolization techniques and clinical outcomes were evaluated using Chi-square or Fisher’s exact tests for categorical variables and Mann–Whitney U tests for non-parametric continuous data, due to the non-normal distribution and small group sizes in several cases.

Logistic regression analysis was performed to identify potential predictors of splenectomy, which represented the main clinical outcome of interest. Independent variables were selected based on clinical relevance and preliminary univariate analyses and included AAST grade, embolization type, presence of vascular injury at CT and DSA, and time from trauma to embolization. Multivariate logistic regression was performed using a complete-case analysis approach: patients with missing values for any of the variables included in the model were excluded from this part of the analysis.

Given the limited number of splenectomy events, a sensitivity analysis was additionally performed using Firth’s penalized logistic regression to account for potential small-sample bias and sparse data structure. This approach improves the reliability of estimates in the context of low event rates. The results of this penalized model are presented in Supplementary Table S2.

All statistical analyses were performed using Python 3.9.7 (libraries: Pandas, SciPy, Statsmodels). A *p*-value < 0.05 was considered statistically significant.

## 3. Results

### 3.1. Patient Population

A total of 281 patients from 11 Italian centers were analyzed. The mean age was 41.3 ± 17.8 years, with a predominance of males (75.4%). The median time from trauma to embolization was 8.5 h (IQR 4.0–17.8), indicating timely management in most cases. In a minority of cases (*n* = 2), embolization was performed more than 72 h after trauma due to delayed complications such as secondary bleeding. These cases were retained in the analysis as they represent real-life clinical scenarios. A comprehensive summary of the demographic and clinical variables is provided in Table 2.

### 3.2. Injury Severity and Imaging Findings

According to the 2018 AAST classification, injury distribution was as follows: grades I–II: 13 (4.6%); grade III: 53 patients (18.9%); grade IV: 149 (53.0%); grade V: 66 (23.5%). Baseline CT identified vascular injuries in 217 (77.2%) cases, whereas angiography confirmed vascular injuries in 256 patients (91.1%). Forty patients (14.2%) underwent embolization despite negative CT findings but had positive DSA results, predominantly among low-grade injuries (AAST I–III). These findings support the role of DSA as a complementary diagnostic tool in cases where CT findings are inconclusive, especially in hemodynamically stable patients with ongoing clinical suspicion.

### 3.3. Embolization Technique and Procedural Details

SAE was categorized as proximal (131 patients, 46.6%), distal (81 patients, 28.8%), and combined proximal–distal (69 patients, 24.6%). The technical success rate was 100%, defined as complete vascular lesion exclusion at post-embolization DSA. Table 3 summarizes the complication distribution and splenectomy rates.

### 3.4. Post-Procedural Outcomes and Complications

A total of 70 patients (24.9%) experienced complications according to CIRSE classification. These included splenic infarction (16 cases, 5.7%), rebleeding (9 cases, 3.2%), and non-target embolization (2 cases, 0.7%). Additionally, splenectomy was performed in 27 patients (9.6%) due to clinical deterioration. However, splenectomy in this context is not regarded as a procedural complication but rather as a failure of NOM, despite technically successful embolization. No statistically significant association was found between embolization technique and splenectomy rate (*p* = 0.526) or between AAST grade and splenectomy (*p* = 0.713), although grade V injuries trended toward higher splenectomy rates.

### 3.5. Multivariate Analysis

**Figure 1:**
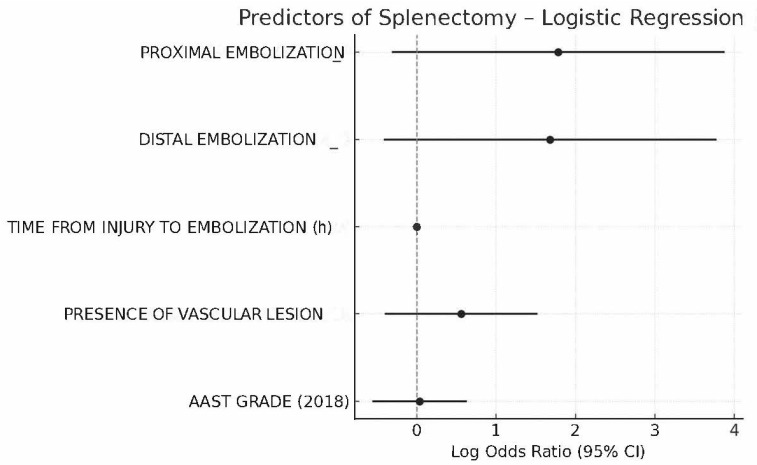
Forest plot of logistic regression analysis showing independent predictors of splenectomy after embolization in patients with blunt splenic trauma. The plot reports the OR and 95% CI for each variable. Although proximal and distal embolization showed higher odds ratios in the regression model, none of the variables—including embolization technique, AAST grade, time to embolization, and presence of vascular lesion—reached statistical significance as independent predictors of splenectomy. The dashed vertical line indicates the null value (OR = 1).

Multivariate logistic regression analysis did not identify any statistically significant independent predictors of splenectomy among the evaluated variables (AAST grade, embolization type, presence of vascular injury at CT and angiography, and time from trauma to embolization). However, patients with higher-grade splenic injuries (AAST IV–V) showed a non-significant trend toward increased odds of requiring splenectomy compared to those with lower-grade injuries (AAST I–III) (odds ratio [OR] 2.11, 95% confidence interval [CI] 0.93–4.38, *p* = 0.074). No relevant trends were observed for embolization technique or timing. These findings are visually summarized in the forest plot presented in Figure 1, which illustrates the odds ratios and confidence intervals for all variables included in the regression model.

### 3.6. Subgroup Analysis

**Figure 2:**
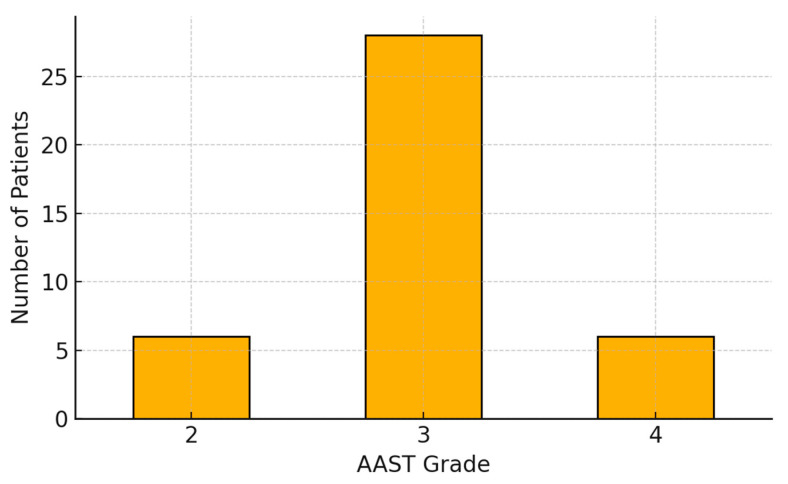
Distribution of AAST grades in the subgroup of patients with negative CT findings but positive angiographic evidence of vascular injury (*n* = 40). The majority of patients had intermediate-grade injuries (AAST III, 70%), while low-grade (AAST II) and high-grade (AAST IV) lesions were less common, each accounting for 15% of the subgroup.

Of 40 patients with negative CT but positive angiographic findings, 34 (85%) had AAST grade II–III injuries, while 6 (15%) had high-grade (AAST IV) lesions. These patients underwent angiography despite the absence of CT-evident vascular injury due to persistent clinical suspicion, such as unexplained hemodynamic instability, hemoglobin drop, or equivocal imaging findings. Four patients (10%) ultimately required splenectomy. The embolization rate in this subgroup was comparable to that of CT-positive patients. The AAST grade distribution within this subgroup is shown in Figure 2, highlighting the diagnostic and therapeutic value of angiography in selected trauma cases with inconclusive CT results.

## 4. Discussion

This large multicenter study offers one of the most extensive real-life evaluations of SAE in Italy to date, providing a robust overview of current interventional strategies for blunt splenic trauma. The findings confirm the widespread adoption of SAE as an effective adjunct to NOM in blunt splenic trauma, particularly for hemodynamically stable patients with moderate-to-severe injuries (AAST grades III–V).

Our results highlight a 100% technical success rate and a relatively low rate of splenectomy (9.6%), in line with previously published data. However, the overall complication rate (24.9%)—including infarction, rebleeding, and splenectomy—emphasizes that SAE, while minimally invasive, is not without risks. As noted in Rasuli et al., complications may be underreported in some series, particularly infarction, which varies in incidence depending on the embolization technique and extent [10].

The high frequency of splenic infarction observed after distal embolization should not be interpreted as a traditional complication. The spleen’s terminal vascularization, particularly at the segmental and subsegmental levels, makes focal infarction a predictable outcome when performing superselective embolization. These areas are usually self-limited and clinically silent, rarely requiring treatment [11,12].

Furthermore, several studies have shown that distal embolization, despite causing partial infarction, is associated with preservation of overall splenic immune function. As demonstrated by Foley et al., patients treated with both proximal and distal embolization had significantly higher levels of circulating IgM memory B cells compared to splenectomized patients, with a trend toward better immunologic preservation in the distal embolization group [8,9,10,11,12,13]. This observation reinforces the concept that infarction of limited parenchymal areas does not compromise the spleen’s immunological role and should not be regarded as an adverse outcome.

In our study, splenectomy was not classified as a procedural complication of splenic artery embolization, but rather as an outcome indicating failure of NOM, consistent with the classification proposed in the WSES guidelines. According to these recommendations, splenectomy following SAE is typically related to persistent or recurrent bleeding rather than technical failure of the embolization procedure itself. This distinction is important to accurately interpret SAE outcomes in real-world trauma settings [14].

A notable aspect of our study was the inclusion of a subgroup of 40 patients who underwent angiography despite the absence of vascular injury on CT. Among these, 85% had intermediate-grade (AAST I–III) injuries, and yet 10% eventually required splenectomy. This subgroup exemplifies a real-life clinical dilemma: persistent clinical signs (tachycardia, hypotension, falling hemoglobin level) may prompt angiography even in the absence of radiological evidence. This practice is supported by Yoo et al., who showed that embolization in CT-negative but high-suspicion trauma patients may improve splenic salvage. Thus, our findings advocate for a selective use of angiography in patients with incongruent clinical and radiological profiles, especially when institutional expertise permits [15].

Another unresolved issue remains the indication for SAE in AAST grade III injuries. While WSES guidelines endorse SAE for grades III–V, many studies still limit embolization to higher grades or clear evidence of vascular injury. Our study included 53 patients with grade III injuries, of whom a substantial number underwent embolization—either due to CT-positive vascular findings or clinical suspicion. Notably, this subgroup achieved high technical success and low splenectomy rates, suggesting that SAE may be a valid therapeutic option even in selected grade III injuries. However, this remains controversial: some authors, like Gasparetto et al. argue that embolization in AAST III should be reserved for cases with contrast blush or pseudoaneurysm, while others, such as Tidadini F et al., support a more liberal approach to prevent delayed bleeding [16,17]. This variability underscores the need for individualized decision-making based on hemodynamic status, imaging, and institutional experience.

Moreover, multivariate analysis in our cohort did not identify statistically significant predictors of splenectomy. Nevertheless, a trend toward increased odds of splenectomy in higher AAST grades was observed, aligning with findings from the literature [18,19]. Embolization type did not influence outcomes, suggesting that technique selection may reflect anatomical and operator factors rather than efficacy [1,20].

Our results are in line with those reported by Gill et al. [21], who confirmed the safety and efficacy of SAE in a Level 1 trauma center setting, with similar splenic salvage rates and complication profiles. Likewise, Lin et al. [22] provided further evidence on the equivalence of proximal, distal, and combined embolization approaches in terms of clinical outcomes, supporting the flexibility of SAE technique selection in real-world trauma care.

The limitations of this study include its retrospective design, heterogeneity in embolization technique and material across centers, and lack of long-term follow-up regarding immunological outcomes or late complications. However, the large sample size, multicenter nature, and inclusion of real-life data provide a valuable contribution to the ongoing debate on optimal SAE strategies in blunt splenic trauma.

In conclusion, our experience demonstrates that splenic artery embolization—particularly when tailored according to vascular injury patterns—is a reliable and effective strategy in the non-operative management of blunt splenic trauma. Its clinical efficacy and safety profile render it comparable to surgical treatment in selected cases, especially in high-grade injuries (AAST grade III and above), which traditionally posed significant management challenges. In light of these results, future guidelines should adopt a three-tiered approach to splenic trauma treatment: (1) NOM, (2) interventional radiological treatment, (3) surgical intervention. In an era of precision medicine, recognizing interventional radiology (IR) as a distinct and primary therapeutic option is crucial to optimize outcomes, reduce unnecessary splenectomies, and preserve organ function in trauma patients. This perspective is increasingly reflected in current trauma management frameworks. The latest WSES guidelines explicitly recommend SAE as a first-line treatment in hemodynamically stable patients with high-grade splenic injuries (AAST III–V) or signs of vascular injury. Similarly, the Eastern Association for the Surgery of Trauma (EAST) and Advanced Trauma Life Support (ATLS) guidelines incorporate embolization into the standard algorithm for blunt splenic trauma [3,23,24]. These positions support the view that IR should be regarded as an autonomous pillar—alongside conservative and surgical approaches—within a modern, tiered treatment strategy for splenic trauma.

## Figures and Tables

**Table 1:** Distribution of patients and demographic characteristics across the 11 participating centers. The order of centers does not reflect relative importance but follows the order of appearance of author affiliations in the manuscript.

Institution	N. Patients	Mean Age ± SD (Years)	Male	Female
Interventional Radiology Department—AORN “A. Cardarelli”, Naples	49	46 ± 20	36	12
Interventional Radiology Unit—Sant’Andrea Hospital of Rome, Rome	8	59 ± 22	3	5
UO Interventional Radiology—A.O.U.P., Pisa	26	47 ± 20	18	8
Radiology Unit—A.O.C. Policlinico di Bari, Bari	9	57 ± 15	8	1
Department of Radiology—AAST Papa Giovanni XXIII, Bergamo	24	43 ± 24	19	5
Radiology Unit, Morgagni-Pierantoni Hospital—AUSL Romagna, Forlì	48	51 ± 21	21	26
Department of Diagnostic Imaging, Oncologic Radiotherapy and Hematology—“Fondazione Policlinico Universitario Agostino Gemelli IRCCS”, Roma	17	56 ± 21	12	5
Department of Diagnostic Imaging and Interventional Radiology—City of Health and Science University Hospital—University of Turin, Turin	36	43 ± 19	27	9
Interventional Radiology Unit—ASST Santi Paolo Carlo, Milano	9	49 ± 27	6	3
Radiology Unit 1—University Hospital Policlinico “G. Rodolico-San Marco”, Catania	7	49 ± 22	4	1
Diagnostic and Interventional Radiology Unit—Maggiore Hospital “C. A. Pizzardi”, Bologna	48	46 ± 20	36	12

**Table 2:** Cumulative summary table of study population.

Parameter	Value
Total number of patients	281
Mean age ± SD (years)	41.3 ± 17.8
Male/Female	191/90
Mean time trauma to embolization (h) ± SD	35.7 ± 80.4
Range of time trauma to embolization (h)	0.5–600.0
AAST grade distribution (III-V)	2: 30 (10.7%); 3: 61 (21.7%); 4: 176 (62.6%); 5: 14 (5.0%)
Embolization type distribution P = proximal embolization; D = distal embolization; C = combined proximal and distal embolization	P: 161 (57.3%); D: 84 (29.9%); C: 36 (12.8%)
Number of complications	70 (24.9%)
Number of splenectomies	27 (9.6%)

**Table 3:** Distribution of complications and splenectomy rates according to embolization technique.

Embolization Type	Total Patients	Splenic Infarction	Rebleeding	Non-Target Embolization	Splenectomy
Combined	36	2	2	2	1
Distal	84	8	9	0	10
Proximal	161	10	4	21	16

## Data Availability

The data presented in this study are not publicly available due to privacy and ethical restrictions, as they contain sensitive clinical information from multiple institutions and were not collected with prior consent for public sharing.

## Supplementary Materials

The following supporting information can be downloaded at: https://www.mdpi.com/article/10.3390/jpm15090420/s1, Table S1: Data collection grid; Table S2: Results of Firth’s penalized logistic regression.
