# Supplementary material for: Real-World Outcomes of Splenic Artery Embolization in Blunt Splenic Trauma: Insights from an Italian Multicenter Cohort
Source: J Pers Med. 2025 Sep 3;15(9):420. doi: 10.3390/jpm15090420 (PMC12470969; doi:10.3390/jpm15090420)
Supplement: Supplementary file 1 [file jpm-15-00420-s001.zip › jpm-3794994-supplementary.pdf]

Supplementary Table S1 (S1)

Name	Surname	Birth date	Age	Sex	Center	Date of trauma	Date of angiography	Time from injury to embolization (	Presence of vascular injury on CT	AAST grade (2018)	WSES grade (2017)	Extent of hemoperitoneum	Presence of vascular injury on angiography	Embolization site	Complications CIRSE classification	Splenectomy	Time of splenectomy from embolization

Supplementary Table S2 (S2)

Variable	OR	CI Lower	CI Upper	p-value
Intercept	0,001694	2,76E-05	0,104067	0,00239
Age	1,012111	0,991882	1,032753	0,242531
Sex	1,827813	0,693618	4,816634	0,222477
AAST	1,246946	0,467496	3,325961	0,659278
moperitone	1,268041	0,857658	1,874791	0,233924
CT_Vascular	0,70601	0,170236	2,92799	0,631456
DSA_Vascula	2,020583	0,736624	5,542524	0,171867
olization_Ty	5,409694	0,668675	43,76537	0,113498
olization_Ty	5,820665	0,707368	47,89606	0,101419
